# Dual-Energy X-Ray absorptiometry associates total body fat and bone mineral content with elevated blood pressure in adult divers

**DOI:** 10.1038/s41598-026-38908-7

**Published:** 2026-04-09

**Authors:** Alex Véliz, Raquel Pereira Berrios, Anita Dörner Paris, Alexis Soto-Salcedo, Cristian Álvarez

**Affiliations:** 1 Social Science Department, University of Los Lagos, Osorno, Chile; 2 Physical Activity and Sports Sciences Department, University of Los Lagos, Osorno, Chile; 3 Departament of Health, University of Los Lagos, Osorno, Chile; 4 School of Psychology, Faculty of Medicine, Mayor University, Temuco, Chile; 5 Exercise and Rehabilitation Sciences Institute, School of Physical Therapy, Faculty of Rehabilitation Sciences, Universidad Andres Bello, Santiago, 7591538 Chile

**Keywords:** Diving, Body composition, Body fat, Bone mineral content, Lifestyle, Blood pressure, Cardiometabolic risk, Cardiology, Diseases, Health care, Medical research, Physiology, Risk factors

## Abstract

To describe the handgrip strength, lifestyle patterns (i.e., physical activity), and body composition of Chilean adult divers of different blood pressure status. Secondly, to associate body fat (BF), fat˗free mass (FFM), and bone mineral content (BMC) with blood pressure. A cross-sectional study was conducted in adult divers men. Participants were categorized into; hypertensive (HTN, *n* = 41), high blood pressure (HBP, *n* = 19), or normotensive (NT, *n* = 35). Primary outcomes were blood pressure, Total and segmental BF %, FFM, and BMC assessed by dual-X absorptiometry. Secondary outcomes comprised handgrip strength (HGS_av_), repetitions in Ruffier test and physical activity. Total and segmental BF % were consistently higher in HTN compared with HBP and NT divers, particularly in total BF (+ 2.8%), arms (BF_A_+2.8%), right arm (BF_RA_ +1.4 to + 3.2%), left arm (BF_LA_ +1.6 to + 2.5%), and trunk (BF_T_ +3.4%; all *P* ≤ 0.05). Age (β = 0.4, [95%CI] 0.1; 0.6, *P* = 0.0004) and total BF % (β = 1.0, [95%CI] 0.1; 0.6, *P* = 0.0004) were significant predictors of SBP in Chilean divers. Total fat-free mass (FFM) does not significantly predict SBP or DBP. Total BMC shows a significant association with SBP (β = − 0.007, [95%CI] − 0.01; 0.0002, *P* = 0.050). Hypertensive Chilean divers show a linear trend to decrease HGS_av_, low cardiovascular fitness, and higher BF than NT peers. There was a significant association between Total BF and Total BMC parameters with both SBP, where segmentally FFM of legs and left leg was associated with both SBP and DBP.

**Supplementary Information:**

The online version contains supplementary material available at 10.1038/s41598-026-38908-7.

## Introduction

High blood pressure is known as arterial hypertension condition (HTN) and is the most common risk factor for cardiovascular disease (CVD), accounting for 22% of CVD in addition with high cholesterol (8%), tobacco (6%), abdominal obesity (6%) and diabetes (5%)^1^. North America demonstrates a higher prevalence and incidence of CVD, but also demonstrate a better control of risk factors and lower mortality compared to Latin America countries, that show lower age-standardized prevalence but increased mortality^1^. In Chile, the HTN show prevalence ~ 26%^2^, but clearly there is a linear trend to increase blood pressure in more adult populations, where adults aged ≥ 60 report around 70% of HTN prevalence. Despite that HTN have been associated with physical inactivity (i.e., lifestyle)^2,3^, in which higher body fat (BF) is a common characteristic in this condition, a current study in adults have proposed that BF was also negatively correlated with bone mineral content (BMC)^4^. Thus, the implementation of health-promotion strategies across different occupational settings plays a critical role in reducing disease risk. For example, frequent interruptions of prolonged sedentary time among office workers have been shown to prevent obesity that could contribute to decrease the development of HTN^5,6^. However, there are other work contexts in which the implementation of early health strategies could also avoid future cardiovascular or bone and frailty conditions (i.e., osteoporosis) due to the work environment.

Diving work produces significant stress on the cardiovascular system due to immersion-induced increased cardiac preload, afterload, and physical exertion, increasing thus the CVD risk^7^. Current diving guidelines have recommended screen cardiovascular health due to 39% of diving fatalities related with cardiac events are reported in divers > 45 years^8^. From here, the cardiovascular risk assessment suggests the inclusion of ECG, and stress tests, coronary calcium scoring and CT angiogram, but only for those who report > 10% of CVD risk^8^. One problematic situation in the diving work is the reduction of the gravitational work environment under the water, in which particularly diver workers dedicated to the aquiculture and mollusk extraction are frequently moving their upper extremity muscles but not the lower limb muscles, turning this segmental of the skeleton a lower impact stimuli^9^, and reducing thus both bone mineralization and muscle mass preservation^10^. Additionally, the maintenance of good physical fitness (i.e., muscle strength or cardiorespiratory fitness) is relevant to optimize diving performance and to prevent CVD as HTN and maintain the appropriate BMC. However, recent information from the Chilean Superintendence of Social Security (SUSESO) in divers of the coast of Chile have reported that 36% of divers declare a smoking habit, 76% alcohol consumption, and 86.7% are classified as overweight/obesity that can negatively impact their physical performance, and cardiovascular health^11^. Unfortunately, there is little information about the lifestyle, and body composition characteristics of divers under different cardiovascular risk, and whether BF, fat-free mass (FFM) and BMC plays a role on blood pressure of diver workers.

Body fat accumulation, particularly BF measured from the trunk region (BF_T_) is widely associated with CVD in adults^12^, and especially in those from an occupational activity of more sedentary behavior including taxi drivers^13^, or office workers^14^. Additionally, it is of wide knowledge, that physiologically, intramyocellular fat accumulation is one of the major mechanisms responsible for type 2 diabetes^15^, and characteristically, diabetic patients show a greater store of BF that increase the CVD risk^16^. Epidemiological studies have also reported that lower physical activity time is associated with increased risk for developing metabolic syndrome, a more complex clinical condition^17^. However, there is little information from cross-sectional studies regarding the association between body composition parameters such as BF, FFM and BMC with blood pressure levels in diver workers. The aim of the present study was to describe the handgrip strength, lifestyle patterns (i.e., physical activity), and body composition of Chilean adult divers of different blood pressure status. Secondly, to associate body fat (BF), fat˗free mass (FFM), and bone mineral content (BMC) with blood pressure. We hypothesize that body composition (total BF, FFM or BMC) or the same outcomes from segmental compartments of adult divers, can be significantly associated with the blood pressure levels of these cohorts.

## Results

### General characteristics

There was significant interaction between HTN, HBP and NT groups, where NT showed lower age vs. HTN (*diff.* ˗12.8 y), and vs. HBP group (*diff.* ˗3.5 y), lower years of diving experience vs. HTN (*diff.* ˗10.6 y), and HBP vs. HTN (*diff.* ˗14.4 y) (Table 1). Similarly, there were significant differences in SBP between NT vs. HTN (*diff*. ˗32.3), NT vs. HBP (*diff.* ˗12.7) and between HBP vs. HTN group (*diff*. ˗19.6 mmHg) (Table 1). DBP was different compared to NT vs. HTN (*diff*. ˗18.4), NT vs. HBP (*diff*. ˗4.8), and between HBP vs. HTN (diff. ˗8.2 mmHg) (Table 1). Pulse pressure was different compared to NT vs. HTN (*diff*. ˗13.0), NT vs. HBP (*diff.* ˗4.8) and between HBP vs. HTN (*diff.* ˗8.2 mmHg) (Table 1). Mean arterial pressure was different compared to NT vs. HTN (*diff*. ˗23.4), NT vs. HBP (*diff*. ˗9.0), and between HBP vs. HTN (diff. ˗14.0 mmHg) (Table 1). The Ruffier index was significantly different among HTN, HBP and NT group at *P* < 0.0001 (Table 1).

**Table 1:** Characteristics of three groups of Chilean diver workers of different blood pressure levels.

Outcomes	Groups			Between-group F; Pvalue; d
	HTN^a^	HBP^b^	NT^c^	
(*n* =)	41	19	35	
Age (y)	59.6 ± 12.2^c^	43.3 ± 13.3^c^	46.8 ± 11.2	***F*(15.6);** ***P*** **< 0.0001; 0.25**
Diving experience (y)	37.9 ± 8.4^c^	23.5 ± 14.0^c^	27.3 ± 12.4	***F*(13.5);** ***P*** **< 0.0001; 0.23**
*Anthropometric*				
Height (m)	168.5 ± 6.1	170.9 ± 8.3	170.5 ± 8.5	*F*(0.97); *P* = 0.382; 0.02
Weight (kg)	84.5 ± 14.6	83.6 ± 9.2	81.3 ± 12.4	*F*(0.57); *P* = 0.562; 0.01
Body mass index (kg·m^2^)	29.0 ± 4.3	27.5 ± 3.5	29.5 ± 3.8	*F*(1.4); *P* = 0.232; 0.03
Normal weight	8	5	4	
Overweight	14	3	14	
Obesity	19	11	17	
*Blood pressure*				
Systolic BP (mmHg)	154.6 ± 10.5^c^	135.0 ± 3.0^c^	122.3 ± 6.4	***F*(152.1);** ***P*** **< 0.0001; 0.76**
Diastolic BP (mmHg)	95.7 ± 11.1^c^	84.4 ± 6.5^ac^	77.3 ± 7.1	***F*(40.6);** ***P*** **< 0.0001; 0.46**
Pulse pressure (mmHg)	58.8 ± 7.1^c^	50.5 ± 6.6^ac^	45.8 ± 6.0	***F*(37.1);** ***P*** **< 0.0001; 0.44**
Mean arterial pressure (mmHg)	115.3 ± 10.3	101.3 ± 4.7^ac^	92.3 ± 5.4	***F*(81.9);** ***P*** **< 0.0001; 0.64**
Heart rate rest (beats/min)	68.0 ± 8.9	66.0 ± 12.2	65.5 ± 8.0	*F*(0.7); *P* = 0.486; 0.01
Basal metabolic rate (kcal/kg)	1580.0 ± 147.6	1524.0 ± 158.2	1561.0 ± 102.5	*F*(1.0); *P* = 0.362; 0.02
*Physical fitness condition*				
*Ruffier test*				
*Ruffier index category*				
Excellent, n=/(%)	6 (14.6)	7 (36.8)	9 (25.7)	***P*** **< 0.0001**^**#**^
Very Good, n=/(%)	28 (68.2)	10 (52.6)	23 (65.7)	
Good, n=/(%)	7 (17.0)	2 (10.5)	3 (8.5)	
Average, n=/(%)	0 (0)	0 (0)	0 (0)	
Poor, n=/(%)	0 (0)	0 (0)	0 (0)	
Very Poor, n=/(%)	0 (0)	0 (0)	0 (0)	
Smoking habit				
Yes, n=/(%)	3 (7.3)	1 (5.2)	4 (11.4)	*P* = 0.543^#^
No, n=/(%)	38 (92.7)	18 (94.8)	31 (88.6)	
Alcohol consumption				
Yes, n=/(%)	41 (100)	19 (100)	32 (91.4)	*P* = 0661^#^
No, n=/(%)	0 (0)	0 (0)	3 (8.6)	
Data are shown as mean and standard error **Groups are described as;** (HTN) Hypertension, (HPB) High blood pressure and (NT) Normotensive group. Data are analyzed by one-way ANOVA at *P* < 0.05. (*d*) Denotes Cohen d effect size at *P* < 0.05. (a) Denotes significantly different vs. HTN group at *P* < 0.05. (c) Denotes significantly different vs. NT group at *P* < 0.05. (#) Data analyzed by X^2^ square test at *P* < 0.05.				

### Lifestyle patterns, muscle strength and cardiovascular fitness

**Fig. 1:**
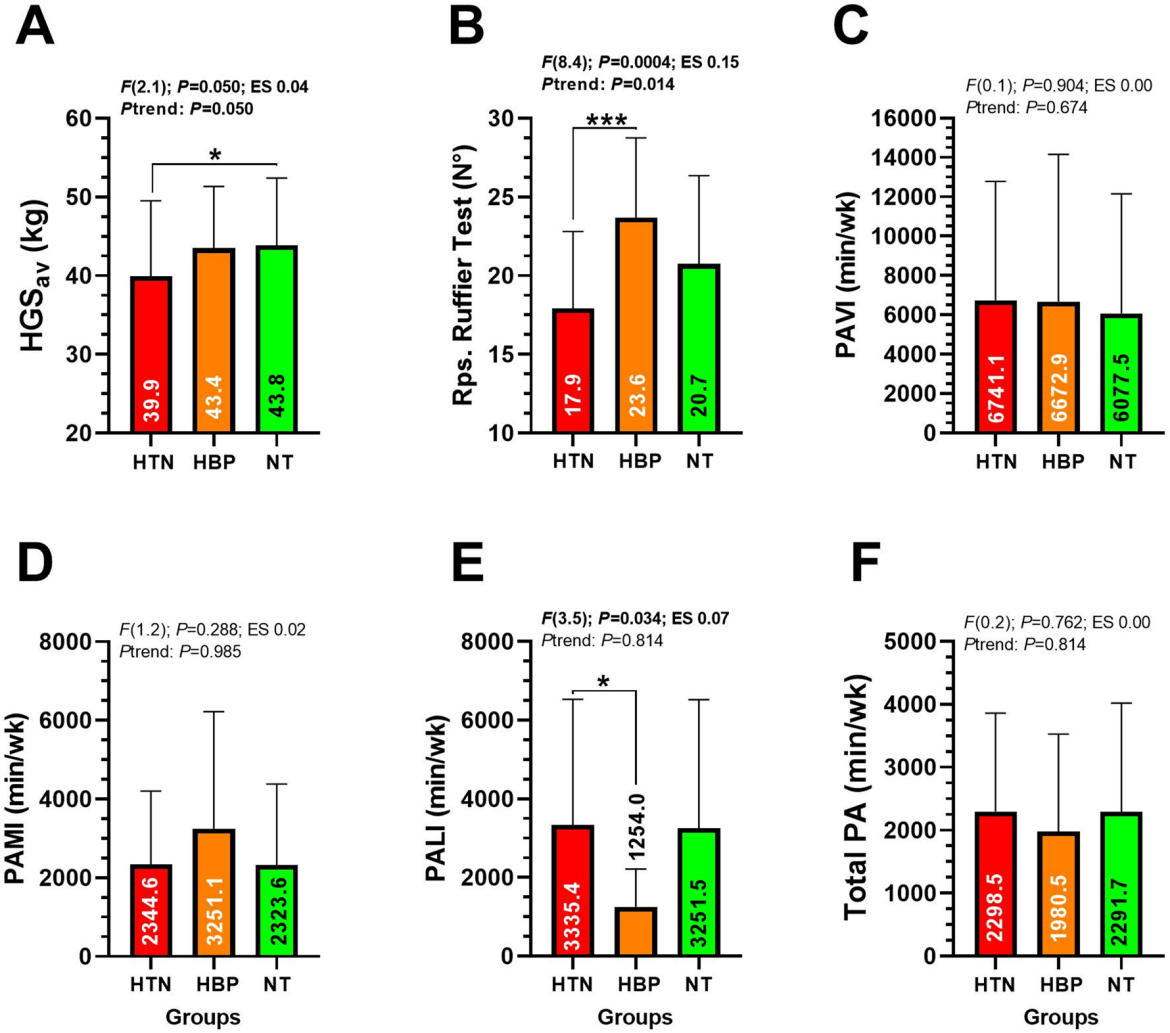
Behavioral and lifestyle characteristics of three groups of Chilean divers of different blood pressure control. Outcomes are described as; (HGSav) Handgrip strength average of both right and left arm. (PAVI) Physical activity of vigorous intensity, (PAMI) Physical activity of moderate-intensity, and (PALI) Physical activity of light-intensity. Groups are described as; (HTN) Hypertension, (HPB) High blood pressure and (NT) Normotensive group. Data are analyzed by one-way ANOVA. (ES) Denotes Cohen’s d effect size measure at P<0.05 level. (Ptrend) Denotes trend of the behavior of the values per group from HTN; HBP; and NT order. (*) Denotes significant differences between categories at P<0.05. (***) Denotes significant differences between categories at P<0.0001.

In the HGS_av_ outcome, there was observed a significant trend to be reduced from NT to HBP and HTN (43.8 ± 8.5, 43.4 ± 7.8 and 39.9 ± 9.5 kg, *P*trend = 0.050), showing HTN divers the lowest HGS_av_ values (panel A). Ruffier Test repetitions were significantly lower in HTN compared to HBP (17.9 ± 4.9 vs. 23.6 ± 5.0 Rps., *P* = 0.0004, *P*trend = 0.014) (Fig. 1 panel B). Additionally, PALI was significantly lower in HBP compared to HTN (1254.0 ± 964.8 vs. 3335.4 ± 3197.0 min/wk, *P* = 0.034), while no significant difference was found between HBP and NT (Fig. 1 panel **E**). No other differences were detected among groups in outcomes PAVI and PAMI.

### Body fat analysis by iDXA

**Fig. 2:**
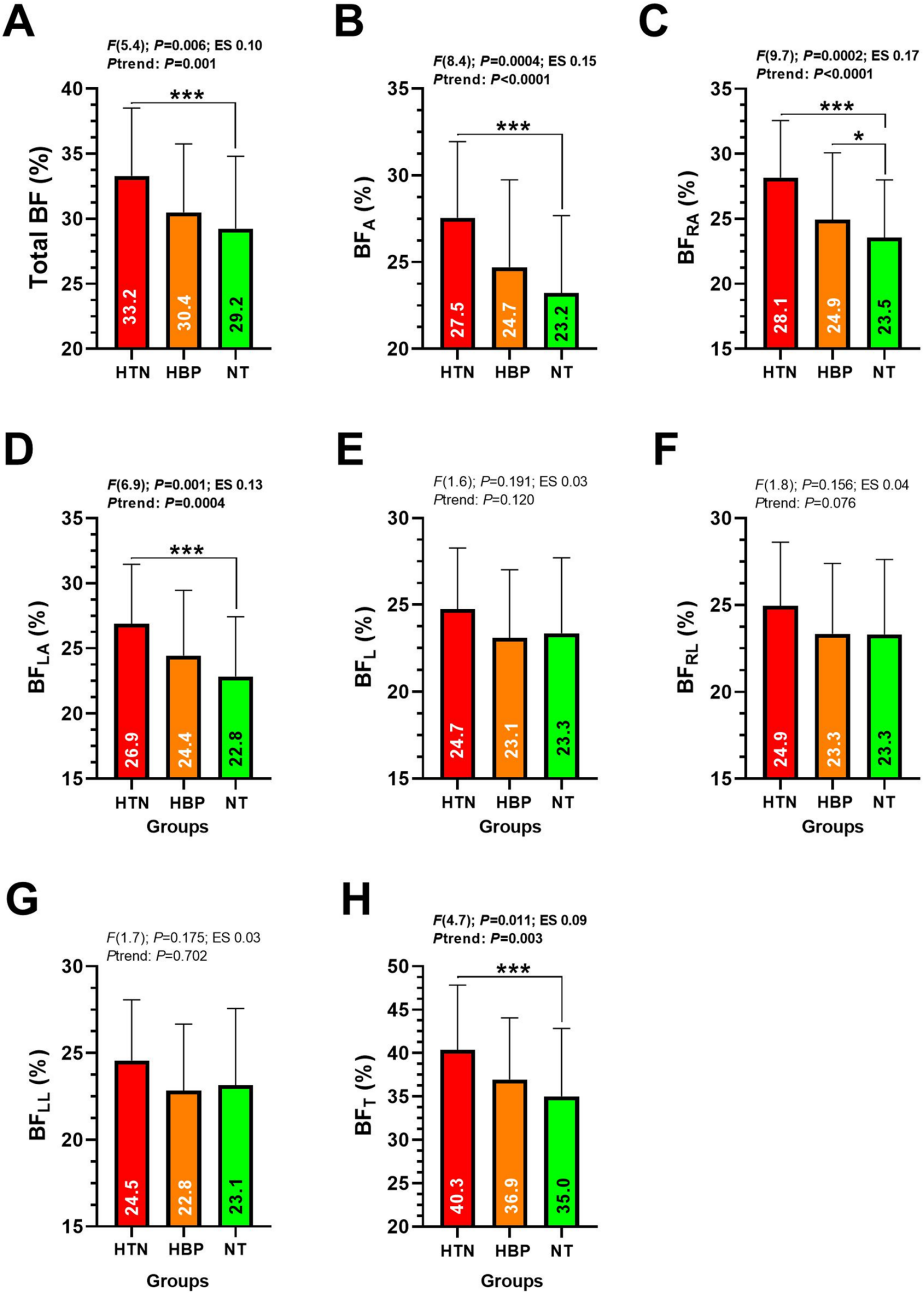
Body composition characteristics by dual X-ray absorptiometry analysis (iDEXA) with respect to bone mineral density (BMC) of type; total BMC (A), arm BMC (B), right arm BMC (C), left arm BMC (D), leg BMC (E), right leg BMC (F), left leg BMC (G) and trunk BMC (H) in Chilean diving workers with different levels of blood pressure. Groups are described as; (HTN) Hypertension, (HPB) High blood pressure and (NT) Normotensive. Data are analyzed by one-way ANOVA. (ES) Denotes Cohen's d effect size measure at P<0.05 level. (Ptrend) Denotes trend of the behavior of the values per group from HTN; HBP; and NT order. (*) Denotes significant differences between categories at P<0.05. (***) Denotes significant differences between categories at P<0.0001.

Total BF percentage was significantly greater in HTN compared to HBP (33.2 ± 5.2 vs. 30.4 ± 5.2%, *P* = 0.006, *P*trend = 0.001) (Fig. 2 panel A). BF in the arms was significantly greater in HTN compared to HBP (27.5 ± 4.1 vs. 24.7 ± 4.6%, *P* = 0.0004, *P*trend < 0.0001) (Fig. 2 panel B). Similarly, BF in the right arm was higher in HTN compared to HBP (28.1 ± 4.3 vs. 24.9 ± 5.1%, *P* = 0.0002, *P*trend < 0.0001), with HBP showing higher values than NT (24.9 ± 5.1 vs. 23.5 ± 4.4%) (Fig. 2 panel C). BF in the left arm was significantly greater in HTN compared to HBP (26.9 ± 4.5 vs. 24.4 ± 5.0%, *P* = 0.001, *P*trend = 0.0004), with HBP again having higher values than NT (24.4 ± 5.0 vs. 22.8 ± 4.5%) (Fig. 2 panel D). Finally, trunk BF was significantly higher in HTN compared to HBP (40.3 ± 7.4 vs. 36.9 ± 7.0%, *P* = 0.011, *P*trend = 0.003) (Fig. 2 panel H).

### Fat˗free mass analysis by iDXA

Among the analyzed variables, only FFM_T_ was significantly lower in HBP compared to HTN (22350.7 ± 8459.0 g vs. 25641.6 ± 4829.0 g, *P* = 0.038), while no significant difference was observed between HBP and NT (Supplementary Material 1: https://figshare.com/s/e1a4bb437c64906eadc1). No other significant differences were found in Total FFM, FFM_A_, FFM_L_, or any of the remaining regional compartments.

### Bone mineral content analysis by iDXA

Among BMC outcomes, there were no differences detected between HTN vs. HBP, or between HBP between NT or between HTN vs. NT group (Supplementary Material 2: https://figshare.com/s/05bcc55bbcb784d093f2).

### Association between total BF, total FFM and total BMC and blood pressure

The regression analysis shows that age was a significant predictor of SBP in Chilean divers (β = 0.4 mmHg·year⁻¹, [95%CI] 0.1; 0.6, *P* = 0.0004) (Table 2). Total BF (%) also has a significant association with SBP (β = 1.0, [95%CI] 0.4; 1.6, *P* = 0.0004), suggesting that higher adiposity is strongly linked to elevated systolic pressure (Table 2). Total fat-free mass (FFM) does not significantly predict SBP (*P* = 0.236), indicating that total muscular mass or lean tissues are not major contributors to blood pressure (Table 2). Total BMC shows a significant association with SBP (β = − 0.007, [95%CI] − 0.01; 0.0002, *P* = 0.050). For DBP models, age shows a significant association with DBP (β = 0.17, [95%CI] 0.007; 0.3, *P* = 0.041) (Table 2). Total BF (%) was also significantly associated with DBP (β = 0.94 mmHg·year⁻¹, [95%CI] 0.5; 1.3, *P* < 0.0001) (Table 2).

**Table 2:** Multiple regression between systolic and diastolic blood pressure with age, and body composition outcomes total body fat, total fat-free mass, and total bone mineral content analyzed by dual X-ray absorptiometry analysis in Chilean divers.

Dependent outcome - Predictor	B (Estimate)	SE	t-test	Pvalue	95%CI
*Systolic blood pressure*					
Intercept	99.3	13.0	7.6	***P*** **< 0.0001**	73.4; 125.2
SBP (mmHg) - Age (y)	0.4	0.1	3.6	***P*** **= 0.0004**	0.1; 0.6
SBP (mmHg) - Total BF (%)	1.0	0.2	3.6	***P*** **= 0.0004**	0.4; 1.6
SBP (mmHg) - Total FFM (g)	0.0001	0.0001	1.1	*P* = 0.236	–0.0001; 0.0004
SBP (mmHg) - Total BMC (g)	–0.007	0.003	1.9	***P*** **= 0.050**	–0.01; 0.0002
*Diastolic blood pressure*					
Intercept	54.22	9.756	5.558	***P*** **< 0.0001**	34.8; 73.6
DBP (mmHg) - Age (y)	0.1709	0.08247	2.073	***P*** **= 0.041**	0.007; 0.3
DBP (mmHg) - Total BF (%)	0.9465	0.2122	4.460	***P*** **< 0.0001**	0.5; 1.3
DBP (mmHg) - Total FFM (g)	0.0001376	9.764e-05	1.409	*P* = 0.162	–5.6; 0.0003
DBP (mmHg) - Total BMC (g)	–0.004522	0.002953	1.531	*P* = 0.129	–0.01; 0.001
Outcomes are described as; (SBP) Systolic blood pressure, (DBP) Diastolic blood pressure, (Total BF) Total body fat, (Total FFM) Total fat˗free mass, and (Total BMC) Total bone mineral content. (B) βeta estimated change in each independent outcome. (SE) Standard error. (*t*-test) Student *t*-test. (95%CI) Denotes 95% confident interval. Bold values denote significant association at *P* ≤ 0.05 level.					

### Association between segmental BF and blood pressure

Age was significantly associated with SBP (β = 0.3 mmHg·year⁻¹, [95%CI] 0.07; 0.5, *P* = 0.012), indicating clearly that SBP increases as age increases (Table 3). None of the regional BF compartments (BF_A_, BF_RA_, BF_LA_, BF_L_, BF_RL_, BF_LL_, or BF_T_ show associations with SBP (Table 3).

**Table 3:** Multiple regression between systolic and diastolic blood pressure and different body fat compartments analyzed by dual X-ray absorptiometry analysis in Chilean divers.

Dependent outcome - Predictor	B (Estimate)	SE	t-test	Pvalue	95%CI
*Systolic blood pressure*					
Intercept	81.03	11.6	6.9	***P*** **< 0.0001**	57.8; 104.2
SBP (mmHg) - Age (y)	0.3	0.1	2.5	***P*** **= 0.012**	0.07; 0.5
SBP (mmHg) - BF_A_ (%)	25.3	31.7	0.7	*P* = 0.427	–37.8; 88.4
SBP (mmHg) - BF_RA_ (%)	–10.8	16.3	0.6	*P* = 0.510	–43.4; 21.7
SBP (mmHg) - BF_LA_ (%)	–15.2	15.4	0.9	*P* = 0.327	–45.8; 15.4
SBP (mmHg) - BF_L_ (%)	–1.4	3.1	0.4	*P* = 0.644	–7.7; 4.8
SBP (mmHg) - BF_RL_ (%)	0.4	2.4	0.1	*P* = 0.858	–4.3; 5.2
SBP (mmHg) - BF_LL_ (%)	–2.5	2.1	1.1	*P* = 0.239	–6.7; 1.7
SBP (mmHg) - BF_T_ (%)	–6.7	3.6	1.8	*P* = 0.065	–13.9; 0.4
*Diastolic blood pressure*					
Intercept	44.0	8.6	5.0	***P*** **< 0.0001**	26.7; 61.3
DBP (mmHg) - Age (y)	0.1	0.09	1.8	*P* = 0.070	–0.01; 0.3
DBP (mmHg) - BF_A_ (%)	18.6	23.6	0.7	*P* = 0.431	–28.3; 65.6
DBP (mmHg) - BF_RA_ (%)	–7.9	12.2	0.6	*P* = 0.517	–32.2; 16.3
DBP (mmHg) - BF_LA_ (%)	–11.4	11.4	0.9	*P* = 0.320	–34.3; 11.3
DBP (mmHg) - BF_L_ (%)	–0.7	2.3	0.3	*P* = 0.747	–5.4; 3.9
DBP (mmHg) - BF_RL_ (%)	2.0	1.7	1.1	*P* = 0.256	–1.5; 5.6
DBP (mmHg) - BF_LL_ (%)	–2.2	1.5	1.4	*P* = 0.152	–5.4; 0.8
DBP (mmHg) - BF_T_ (%)	–2.1	2.6	0.7	*P* = 0.434	–7.4; 3.2
Outcomes are described as; (SBP) Systolic blood pressure, (DBP) Diastolic blood pressure, (BF_A_) Body fat arms, (BF_RA_) Body fat right arm, (BF_LA_) Body fat left arm, (BF_L_) Body fat legs, (BF_RL_) Body fat right leg, (BF_LL_) Body fat left leg, (BF_T_) Body fat trunk. (B) βeta estimated change in each independent outcome. (SE) Standard error. (*t*-test) Student *t*-test. (95%CI) Denotes 95% confident interval. Bold values denote significant association at *P* ≤ 0.05 level.					

### Association between segmental FFM and blood pressure

Age shows a strong and significant positive association with SBP (β = 0.4 mmHg·year⁻¹, [95%CI] 0.2; 0.7, *P* = 0.0003) (Table 4). There were significant association between FFM_L_ with SBP (β = 0.03, [95%CI] − 0.0001; 0.06, *P* = 0.050), and between FFM_L_ (β = − 0.03, [95%CI] − 0.06; 0.001, *P* = 0.050), and FFM_LL_ (β = − 0.03, [95%CI] − 0.06; 0.0008, *P* = 0.056) (Table 4).

**Table 4:** Multiple regression between systolic and diastolic blood pressure and different fat-free mass compartments analyzed by dual X-ray absorptiometry analysis in Chilean divers.

Dependent outcome - Predictor	B (Estimate)	SE	t-test	Pvalue	95%CI
*Systolic blood pressure*					
Intercept	104.8	11.4	9.1	***P*** **< 0.0001**	81.9; 127.6
SBP (mmHg) - Age (y)	0.4	0.1	3.8	***P =*** **0.0003**	0.2; 0.7
SBP (mmHg) - FFM_A_ (g)	–0.005	0.04	0.1	*P =* 0.894	–0.08; 0.07
SBP (mmHg) - FFM_RA_ (g)	0.009	0.04	0.2	*P =* 0.827	–0.07; 0.09
SBP (mmHg) - FFM_LA_ (g)	0.004	0.04	0.1	*P =* 0.911	–0.07; 0.08
SBP (mmHg) - FFM_L_ (g)	0.03	0.01	1.9	***P =*** **0.051**	–0.0001; 0.06
SBP (mmHg) - FFM _RL_ (g)	–0.03	0.01	1.9	*P =* 0.060	–0.06; 0.001
SBP (mmHg) - FFM_LL_ (g)	–0.03	0.01	1.9	***P =*** **0.056**	–0.06; 0.0008
SBP (mmHg) - FFM_T_ (g)	–0.0003	0.0009	0.3	*P =* 0.700	–0.002; 0.001
*Diastolic blood pressure*					
Intercept	63.0	8.7	7.1	***P*** **< 0.0001**	45.5; 80.4
DBP (mmHg) - Age (y)	0.2	0.09	2.2	***P =*** **0.030**	0.02; 0.4
DBP (mmHg) - FFM_A_ (g)	–0.008	0.03	0.2	*P =* 0.780	–0.07; 0.05
DBP (mmHg) - FFM_RA_ (g)	0.008	0.03	0.2	*P =* 0.798	–0.05; 0.07
DBP (mmHg) - FFM_LA_ (g)	0.007	0.03	0.2	*P =* 0.804	–0.05; 0.07
DBP (mmHg) - FFM_L_ (g)	0.02	0.01	2.0	***P =*** **0.046**	0.0005; 0.05
DBP (mmHg) - FFM _RL_ (g)	–0.02	0.01	1.8	*P =* 0.070	–0.05; 0.002
DBP (mmHg) - FFM_LL_ (g)	–0.02	0.01	2.0	***P =*** **0.042**	–0.04; 0.0008
DBP (mmHg) - FFM_T_ (g)	0.0001	0.0006	0.1	*P =* 0.865	–0.001; 0.001
Outcomes are described as; (SBP) Systolic blood pressure, (DBP) Diastolic blood pressure, (FFM_A_) Fat˗free mass arms, (FFM_RA_) Fat˗free mass right arm, (FFM_LA_) Fat˗free mass left arm, (FFM_L_) Fat˗free mass legs, (FFM_RL_) Fat˗free mass right leg, (FFM_LL_) Fat˗free mass left leg, (FFM_T_) Fat˗free mass trunk. (B) βeta estimated change in each independent outcome. (SE) Standard error. (*t*-test) Student t-test. (95%CI) Denotes 95% confident interval. Bold values denote significant association at *P* ≤ 0.05 level.					

For DBP, age shows a significant association (β = 0.2 mmHg·year⁻¹, [95%CI] 0.02; 0.4, *P* = 0.030) (Table 4). Similarly, Total FFM (FFM_L_) was significantly associated with higher DBP (β = 0.02, [95%CI] 0.0005; 0.05, *P* = 0.046) (Table 4). Left leg FFM_LL_ shows a significant inverse association (β = − 0.02, [95%CI] − 0.04; − 0.0008, *P* = 0.042) (Table 4).

### Association between segmental BMC and blood pressure

(Table 5) summarizes the results of multiple linear regression analyses evaluating the associations between SBP and DBP and different BMC compartments measured by iDXA technology in Chilean adult divers. For SBP, age showed to be a significant independent predictor (β = 0.5, [95%CI] 0.2 to 0.7, *P* < 0.0001;), indicating higher SBP values with increasing age. Beyond the full regression model in SBP, none of the segmental or total BMC compartments, including arms, legs, trunk, or bilateral segments, showed significant associations with SBP (Table 5). Similarly, for DBP, age was positively associated with DBP (β = 0.2 mmHg·year⁻¹, [95%CI] 0.03 to 0.4, *P* = 0.022), whereas total and segmental BMC measures were not significant predictors (Table 5).

**Table 5:** Multiple regression between systolic and diastolic blood pressure and different bone mineral content compartments analyzed by dual X-ray absorptiometry analysis in Chilean divers.

Dependent outcome - Predictor	B (Estimate)	SE	t-test	Pvalue	95%CI
*Systolic blood pressure*					
Intercept	76.7	19.5	3.9	***P =*** **0.0002**	37.0; 115.5
SBP (mmHg) - Age (y)	0.5	0.1	4.2	***P*** **< 0.0001**	0.2; 0.7
SBP (mmHg) - BMC_A_ (g)	0.3	0.2	1.2	*P =* 0.219	–0.2; 0.9
SBP (mmHg) - BMC_RA_ (g)	–0.2	0.2	0.7	*P =* 0.426	–0.7; 0.3
SBP (mmHg) - BMC_LA_ (g)	–0.3	0.3	0.9	*P =* 0.363	–1.1; 0.4
SBP (mmHg) - BMC_L_ (g)	–0.003	0.009	0.3	*P =* 0.745	–0.02; 0.01
SBP (mmHg) - BMC_RL_ (g)	–0.06	0.1	0.6	*P =* 0.512	–0.2; 0.1
SBP (mmHg) - BMC_LL_ (g)	0.05	0.09	0.6	*P =* 0.531	–0.1; 0.2
SBP (mmHg) - BMC_T_ (g)	0.01	0.01	0.8	*P =* 0.401	–0.01; 0.04
*Diastolic blood pressure*					
Intercept	48.5	15.4	3.1	***P =*** **0.002**	17.8; 79.1
DBP (mmHg) - Age (y)	0.2	0.09	2.3	***P =*** **0.022**	0.03; 0.4
DBP (mmHg) - BMC_A_ (g)	0.1	0.2	0.7	*P =* 0.485	–0.2; 0.6
DBP (mmHg) - BMC_RA_ (g)	–0.1	0.2	0.5	*P =* 0.609	–0.5; 0.3
DBP (mmHg) - BMC_LA_ (g)	–0.1	0.3	0.6	*P =* 0.518	–0.8; 0.4
DBP (mmHg) - BMC_L_ (g)	–0.003	0.007	0.4	*P =* 0.665	–0.01; 0.01
DBP (mmHg) - BMC_RL_ (g)	–0.0003	0.07	0.004	*P =* 0.996	–0.1; 0.1
DBP (mmHg) - BMC_LL_ (g)	0.02	0.07	0.2	*P =* 0.769	–0.1; 0.1
DBP (mmHg) - BMC_T_ (g)	0.01	0.01	1.2	*P =* 0.221	–0.008; 0.03
Outcomes are described as; (SBP) Systolic blood pressure, (DBP) Diastolic blood pressure, (BMC_A_) Bone mineral content arms, (BMC_RA_) Bone mineral content right arm, (BMC_LA_) Bone mineral content left arm, (BMC_L_) Bone mineral content legs, (BMC_RL_) Bone mineral content right leg, (BMC_LL_) Bone mineral content left leg, (BMC_T_) Bone mineral content trunk. (B) βeta estimated change in each independent outcome. (SE) Standard error. (*t*-test) Student *t*-test. (95%CI) Denotes 95% confident interval. Bold values denote significant association at *P* ≤ 0.05 level.					

## Discussion

The aim of this study was to describe the handgrip strength, lifestyle patterns (i.e., physical activity), and body composition of Chilean adult divers of different blood pressure status. Secondly, to associate BF, FFM, and BMC with blood pressure. The main study results yielded were (i) Chilean divers classified as hypertensive report a linear trend of a decreased muscle strength HGS_av_ (Fig. 1), (ii) a lower cardiovascular fitness (i.e., by Ruffier test), higher BF (i.e., Total BF, BF_A_, and BF_T_) in comparison with normotensive peers, revels a (iii) significant association among Total BF percentage and Total BMC with particularly SBP (Table 2), and (iv) segmental body composition show also that specifically FFM_L_ and FFM_LL_ (i.e., lower limb skeletal muscle indicators) are associated with SBP and DBP in Chilean adult diver workers. These results summarize that characteristically muscle strength (i.e., HGS_av_) and cardiovascular recovery (i.e., Ruffier test) are two key markers of physical fitness that are reduced in divers with hypertension, and that Total BF % and BMC play a role in the blood pressure behavior. These individuals also exhibited a higher burden of cardiovascular risk factors, with increased BF in 5 out of 8 regions assessed by the iDXA analyses compared with their normotensive peers (Fig. 2), and with similar lifestyle characteristics between HTN and normotensive divers (i.e., PAVI, PAMI, and in the groups despite age differences (Table 1).

About our first results, the present study revealed that HTN divers tend to exhibit a reduced HGS_av_ (*diff.* ˗3.5 kg vs. HBP groups, and *diff.* ˗3.9 kg vs. NT group) (Fig. 1A). This finding aligns with previous studies showing that HTN is frequently associated with diminished physical function and muscular performance, particularly in older adults. In brief, Mainous et al.^18^ from the American National Health and Nutrition Examination Survey (NHANES) study, reported that in adults aged ≥ 20 years old in (*n* = 1467) subjects with normal BMI and handgrip strength were lower in participants with HTN (56.3 kg) compared with those classified as normotensive (66.0 kg). Thus, considering the physical demands of diving work, particularly under environments involving repetitive upper-limb movements as with aquiculture/mollusk extraction, a reduced HGS_av _may not only impact work performance but also increase the individual’s cardiovascular risk. For example, Lera et al^19^. reported from (*n* = 1956) Chilean adults, that the cut-off point to the 5^th^, 10^th^, and 25^th^ percentile of HGS was of 22.0, 25.7 and 30.4 kg to men of 60 to 64.9 years.

About our second results, divers classified as HTN demonstrated lower cardiovascular fitness when we examined performance (i.e., by Repetitions during knee flexion/extension) during the Ruffier test (Fig. 1) along with higher BF% compared to HBP or NT divers. In parallel, despite our results do not showed major Total or segmental FFM differences among the different blood pressure categories of divers (Supplementary Material 1: https://figshare.com/s/e1a4bb437c64906eadc1), this lower functional capacity shown by those in HTN that are ~ 60 y aged (Table 1) reveal an alert of future potential reduced skeletal muscle mass and thus a sarcopenic condition which is more related with the frailty condition described in older adults^20^. By contrast, it is well known that cardiorespiratory fitness, muscle strength and lower limb function are associated with the work ability, but when these capacities are declined there is also a reduced work ability^21^. Thus, if the natural gravitational environment of divers promotes a reduced lower limb and skeletal muscle mass stimuli, there is a need to increase the prevention of the declined muscle function and the work ability by promoting specific muscle health promotion or programs in some specific work contexts such as diving. Other explanation about these differences could suggest a more compromised muscular/functional condition in divers with HTN, that could be a result more influenced by the sample size of the study more than the characteristic of the sample. On the other hand, considering that diving environment exposes the cardiovascular system to higher strain due to immersion, physical exertion, and altered hydrostatic pressures^22^, individuals with poorer cardiovascular recovery and lower FFM may face an elevated risk of cardiovascular events. Our findings support the need for early identification of at-risk individuals, with more need of increasing physical activity and preserving muscle mass to mitigate the physiological consequences of high blood pressure and working under the water by hours. Furthermore, a significant association was found between segmental BF distribution and both SBP/DBP, particularly with BF_T_, BF_A_, and Total BF, as measured by iDXA. These results confirm our initial hypothesis that BF_T_ could be related to playing a key role in the elevation of blood pressure among divers. This is in coherence with broader epidemiological evidence showing visceral BF as a major contributor to HTN and cardiovascular disease^23^. The localized body fat accumulation as BF_T_ and B_FA_, often associated with metabolic dysfunction and insulin resistance^24^, appears to be particularly impactful for HTN divers. Worryingly, a recent governmental study by the Superintendence of Social Security (SUSESO) reported that 36% of divers report having a smoking habit, and 76% consume alcohol occasionally, and ~ 4 to 5% have reported a bone fracture as a main accident^11^. Chilean divers also report to have a 86.7% overweight/obesity prevalence, increasing their risk for cardiovascular disease^11^.

In our third result, in spite of we do not observed differences among blood pressure categories in BMC (Supplementary Material 2: https://figshare.com/s/05bcc55bbcb784d093f2), a significant association was found between Total BF % with SBP and DBP. Zhang et al.^25^ in a study of (*n* = 14412) participants from the National Health and Nutrition Survey using similar iDXA analyses (aged ~ 36 y), reported that Total BF %, total muscle mass, and BF_T_ were associated with SBP, but after adjustments BF_T_, BF_L_ and muscle mass were the major determinants outcomes to the SBP behavior. Park et al.^26^ reported that from (*n* = 4864) Korean participants that were divided into quintiles, and the results indicated that compared with quintile 1 (Control normotensive), quintile 4 and 5 showed a significant elevated Total BF % than quintile 1. Applying a similar quintile strategy, we have observed similar results in relationship with these authors, showing quintile 4 (Total BF 34.1%) and 5 (Total BF 38.4%) of categorized HTN divers more Total BF % in comparison with Q1 (Total BF 23.3%), and this situation was similar in the Total BF of the HBP and NT category of divers through quintiles (Supplementary Material 3: https://figshare.com/s/50c63812839017439890). Thus, considering that those in HTN and NT groups reported similar Ruffier test repetitions (mean: 19.3 Repetitions.) and similar patterns of PAVI, PAMI and PALI (Fig. 2), it is relevant in future studies to include more complex physical fitness parameters, such as cardiovascular, vascular, metabolic and cardiorespiratory fitness outcomes in combination with biomarkers to characterize better the specific occupational context as the diving work. In this third result, BMC also showed to be associated with SBP (Table 2). Previous studies have established a relationship between high blood pressure and osteoporosis in HTN older adult populations^27^, however, in middle-aged adult males (i.e., ~ 50 y), hypertensive males have been also reported a low bone mineral density (i.e., BMC to our study)^28^. Worryingly, other evidence has shown that individuals with low bone mineral density also report a higher pulse wave velocity, a known marker of arterial stiffness and atherosclerosis^29^, that increase the need of future studies to solve the role of total and segmental body composition, physical fitness and lifestyle characteristics at the level of blood pressure under unusual physiological environmental such as the diving work.

Lastly, our fourth result revealed that FFM_L_ and FFM_LL_ (i.e., lower limb skeletal muscle indicators) were associated with SBP and DBP, that are more commonly reported results. Ittermann et al.^30^ described in (*n* = 4467) adults (i.e., not taking hypotensive pharmacotherapy) aged 21 to 82 y, that increasing BF and FFM (i.e., measured by electrical bio-impedance) has a substantial impact of the development or reversion of HTN in adults being not at all clear their findings. Other studies including (*n* = 5058) Japanese men > 20 y from Takase et al.^31^ reported that those subjects with highest BF and lowest FFM showed a significant association with HTN, however, there is a need in future studies of add the skeletal muscle mass. Thus, routine iDXA measurement of segmental body composition analyses may offer valuable insights together with the blood pressure quantification for future stratification and prevention of major health risks associated with the professional diving work. Jepson et al.^8^ noted that due to the diving work stress on the cardiovascular system, immersion-induced increased cardiac preload, afterload, and physical exertion, divers aged ≥ 45 years or older should have a cardiovascular health screening by ECG, exercise stress test, or more complex testing for reporting on the increased cardiovascular risk.

The present study did present with limitations, for example (i) we did not measured the physical activity levels by direct accelerometry equipment’s, however, we used a widely known GPAQv2 physical activity questionnaire applied in adults populations of different characteristics, (ii) blood pressure was measured in groups of 3 participants, that could decrease the quiet conditions required by each participant to show more real resting blood pressure values, and the blood pressure was measured with an automated cuff device which have not been validated in clinical settings with a standard sphygmomanometer, (iii) the heart rate during the Ruffier test (HR1, HR2, HR3) were measured by pulse finger detection and using a hand watch, but not by a cardiometer, (iv) the NT and HBP groups were younger (i.e., ~ 10 y) than the HTN group, which could partly explain their lower HGS_av_ and fewer repetitions in the Ruffier test, however, despite this age difference, the groups exhibited similar physical activity patterns. In studies of this kind, especially in a group as rarely examined as these diver workers R² values around 10–20% are usually taken as meaningful contributions to the explained variance. However, the present study also contains some strengths such as (i) body composition was analyzed by a validated robust iDXA equipment with results possible to extrapolate for future comparisons, (ii) the present study contains not only body composition but also valuable socio-demographic information, and (iii) all participants were divers working actively in the diving of mollusk of the reported cities in the Los Lagos region, coast of Chile, being possible to contact them for future monitoring.

## Conclusion

Chilean divers in hypertensive condition of blood pressure display overall physical activity levels comparable to their normotensive counterparts, yet they present lower HGS_av_, reduced cardiovascular fitness as reflected by the Ruffier test, and a higher Total BF percentage. Beyond the influence of age into the regression models, Total BF % and Total BMC showed significant associations with SBP. In contrast, the segmental analyses indicated that FFM in the legs but particularly the left leg, was associated to both SBP and DBP in adult male divers.

## Methods

### Participants and study design

This corresponds to a descriptive cross-sectional study developed by the Universidad de Los Lagos, Puerto Montt, Chile and collaborators. The study was conducted from June to September 2024, and participated adult divers from Puerto Montt, Calbuco, Maullín and Ancud cities of the Region de Los Lagos, the coast of Chile. The participants were invited to participate in the study by each social diving group in which they were associated in these cities. The study was approved by the Universidad Mayor Ethical Committee (approval N° 0492). All participants signed an informed consent before being involved in the study, and the study was carried out following the Helsinki Declaration for human studies.

Criteria of inclusion: (i) adult diver, (ii) to be member of a diver association group of the cities of Puerto Montt, Calbuco, Maullín or Ancud (Chile), (iii) declare availability of 2 days to perform the test and body composition analysis, (iv) diver experience of at least 5 years of continuum activity and (v) to provide your updated medical information of the last 3 months (cardiovascular health program control card), if applicable. Criteria of exclusion were: (i) unable to understand the instructions for each test, (ii) psychomotor alterations that make it difficult to stand still during 10 minutes in the iDXA measurement, (iii) not be a peacemaker patient or use other electronic equipment for health such as insulin pumps. The final sample included groups of diving employees of different blood pressure levels such as; arterial hypertension (HTN, *n* = 41, BMI), with elevated blood pressure (HBP, *n* = 19, BMI) and normotensive divers (NT, *n* = 35, BMI). The CONSORT study design can be seen in (Supplementary Material 4: https://figshare.com/s/b26e847c8e7a2a510ea5).

### Lifestyle

#### Physical activity patterns

The vigorous (PAVI), moderate (PAMI) and light physical activity (PALI) were measured using the “Global Physical Activity Questionnaire version 2” (GPAQv2)^32^ and this was used to assess sedentary behavior. All evaluators received prior guidance from the research team, and the assessments were carried out by the main investigators in a quiet, comfortable setting. Additional outcomes collected were smoking habits and alcohol consumption. These identified the current smoker (daily and occasionally [i.e., smoke only in environments of social activities]) and the ex-smoker, allowing to obtain the number of cigarettes smoked and the persistence of the habit, but to this study the information was registered only as non-smoker and smoker^33^.

### Muscle strength condition

The muscle strength was evaluated by the HGS, where both hands were used in three attempts in a seated position, and we measured in three attempts the handgrip of the right and left arm, registered the average of both arms (HGS_av_). These measurements were developed by using a digital handheld dynamometer (Jamar^®^, PLUS+, Sammons Preston, Patterson Medical, Illinois, United States) following previous studies in adults^34^.

#### Cardiorespiratory fitness condition

All the subjects evaluated were instructed in the following way: firstly, the heart rate at rest was measured in a standing position for 15 s multiplied by 4 to know the equivalent beats per minute, recorded as (HR1), then the person stood up, did leg flexion-extensions at a steady pace with the throne straight at 90° of knee flexion, raising arms in front of the front while flexing for 45 s. Immediately after performing the leg flexion-extensions exercise, the heart rate (HR2) is taken and recorded again, followed by a 1-minute rest in a seated position, and finally the heart rate (HR3) is recorded again. The results are then interpreted using the following formula, Ruffier index: [(HR1 + HR2+HR3) ˗ 200]/100, where the aerobic endurance is determined according to the following values [0 ‘very good performance’, 0.1–5.1 ‘good performance’, 5.1–10 ‘average performance’, > 10–15 ‘insufficient performance’, 15.1–20 ‘poor performance’ and (requires medical evaluation).

### Anthropometry (secondary outcomes)

Weight was measured with a BIA equipment InBody120™ scale (tetrapolar 8-point tactile electrode system, model BPM040S12F07, Biospace, Inc., Seoul, Korea) with at 0.1 kg precision following previous studies^35^. Height was measured with a SECA™ 213-Topmedic portable stadiometer (Germany). BMI was calculated using weight and height squared.

#### Blood pressure measurement (Main outcomes)

Blood pressure was measured on three attempts with rest intervals of at least 1 min between measurements, using digital cuff instrument positioned in the arm OMRON™ (Model HEM-7142, United States) validated clinically and previously used in other hypertensive American countries^36^. Blood pressure was categorized according to the criteria of the European Society of Cardiology into hypertension (HTN; SBP ≥ 140 or DBP ≥ 90 mmHg), high blood pressure (HBP; SBP 130–139 mmHg or DBP 85–89 mmHg), and normal blood pressure or normotensive (NT; SBP 130–139 mmHg or DBP 85–89 mmHg)^37^.

#### Body composition measurement (Main outcomes)

All participants were screened in their body fat in the following outcomes; total body fat (Total BF), body fat of the arms (BF_A_), body fat right arm (BF_RA_), body fat left arm (BF_LA_), body fat of the legs (BF_L_), body fat of the right leg (BF_RL_), body fat of the left leg (BF_LL_), and body fat of the trunk (BF_T_). Similarly, fat˗free mass was analyzed in the following outcomes; total fat˗free mass (Total FFM), fat˗free mass of the arms (FFM_A_), fat˗free mass of the right arm (FFM_RA_), fat˗free mass left arm (FFM_LA_), fat˗free mass of the legs (FFM_L_), fat˗free mass of the right leg (FFM_RL_), fat˗free mass of the left leg (FFM_LL_), and fat˗free mass of the trunk (FFM_T_). Also, the bone mineral content was analyzed and reported by the following outcomes; total bone mineral content (Total BMC), bone mineral content of the arms (BMC_A_), bone mineral content of the right arm (BMC_RA_), bone mineral content of the left arm (BMC_LA_), bone mineral content of the legs (BMC_L_), bone mineral content of the right leg (BMC_RL_), bone mineral content of the left leg (BMC_LL_), and bone mineral content of the trunk (BMC_T_). All measurements were developed in the Lunar iDXA equipment (Healthcare General Electric Company, ENCORE 18 Software, United States). The participants arrived at the Universidad de Los Lagos laboratory from Monday to Friday (9 to 13:00 pm) in the morning. Before the measurement a personal interview was developed to check conditions for the scanning analyses. During the iDXA measurement, all participants were in supine position with light clothing, without shoes or metal objects or jewelry. The iDXA measurements and the characteristics of the diver equipment and diving conditions can be seen from online (Supplementary Material 5: https://figshare.com/s/3bff32e9b7ff2c0ed29a).

### Statistical analysis

Data are shown as mean and (±) standard deviation for continuous variables and as frequency (*n* =) and percentage (%) for categorical variables. To test differences between groups, One-way ANOVA and Tukey’s post hoc post hoc test for multiple comparisons between groups at *P* < 0.05 alpha error level was applied. For nonparametric variables, the Kruskal-Wallis test or REML test was applied for mixed models of analysis, and Dunn’s post hoc for multiple comparisons at *P* < 0.05 alpha error level. Additionally, Cohen’s *d* effect size test was applied at *P* < 0.05 level. Multiple regression analyses were carried out using body composition outcomes (Total BF, FFM, BMC and their different segmental variations) as dependent outcomes with (SBP or DBP) as independent outcome and considering their group-differences adjusted by the age variable, to test the association between body composition and blood pressure. These analyses were performed using Grap Pad Prism v. 8.0 statistical software (Chicago, Illinois, United States). Additionally, using the SPSS™ software 24 version for Windows (IBM-SPSS Inc., Chicago, IL, USA), quintiles of Total BF % were calculated by the different HTN, HBP and NT groups to reveal potential quintile-differences in this outcome.

## Supplementary Information

Below is the link to the electronic supplementary material.

Supplementary Material 1

Supplementary Material 2

## Data Availability

The datasets generated and/or analyzed during the current study are available from the online site in (**Supplementary Material 6** : [**https://figshare.com/s/19365f55a3c4360770b7**](https:/figshare.com/s/19365f55a3c4360770b7))
