# Supplementary material for: Dual-Energy X-Ray absorptiometry associates total body fat and bone mineral content with elevated blood pressure in adult divers
Source: Sci Rep. 2026 Apr 9;16:11783. doi: 10.1038/s41598-026-38908-7 (PMC13065840; doi:10.1038/s41598-026-38908-7)
Supplement: Supplementary file 2 — Supplementary Material 2 [file 41598_2026_38908_MOESM2_ESM.pdf]

Supplementary Material

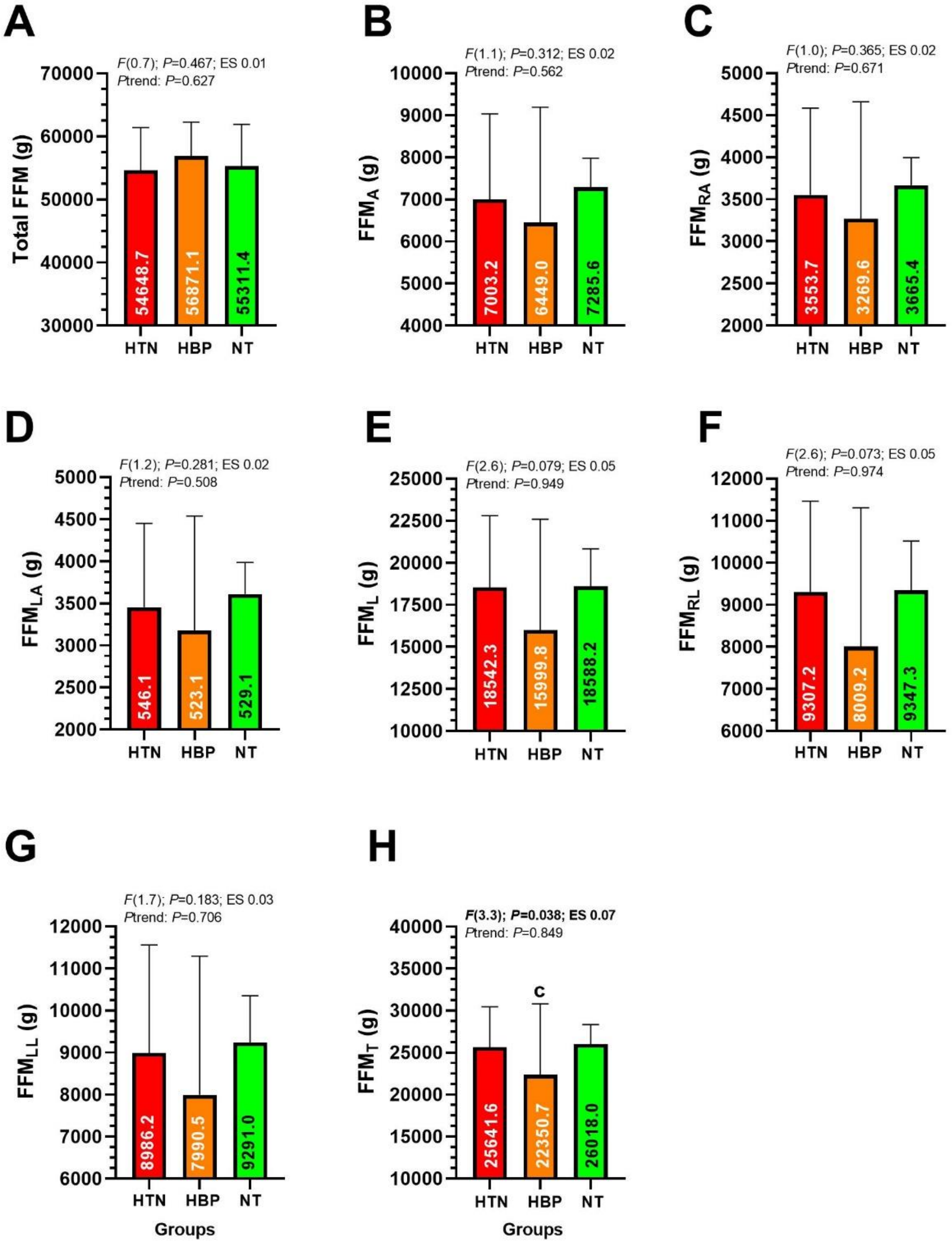

(Supplementary Material 1). Body composition characteristics by dual X-ray absorptiometry analysis (iDXA) with respect to fat free mass (FFM) of type; total FFM (A), arm FFM (B), right arm FFM (C), left arm FFM (D), leg FFM (C), right leg FFM (F), left leg FFM (G) and trunk FFM (H) in Chilean diving workers with different levels of blood pressure. Groups are described as; (HTN) Hypertension, (HPB) High blood pressure and (NT) Normotensive group. Data are analyzed by one-way ANOVA. (ES) Denotes Cohen's d effect size measure at $P<0.05$ level. (*P*trend) Denotes trend of the behavior of the values per group from HTN; HBP; and NT order. (*) Denotes significant differences between categories at $P<0.05$.

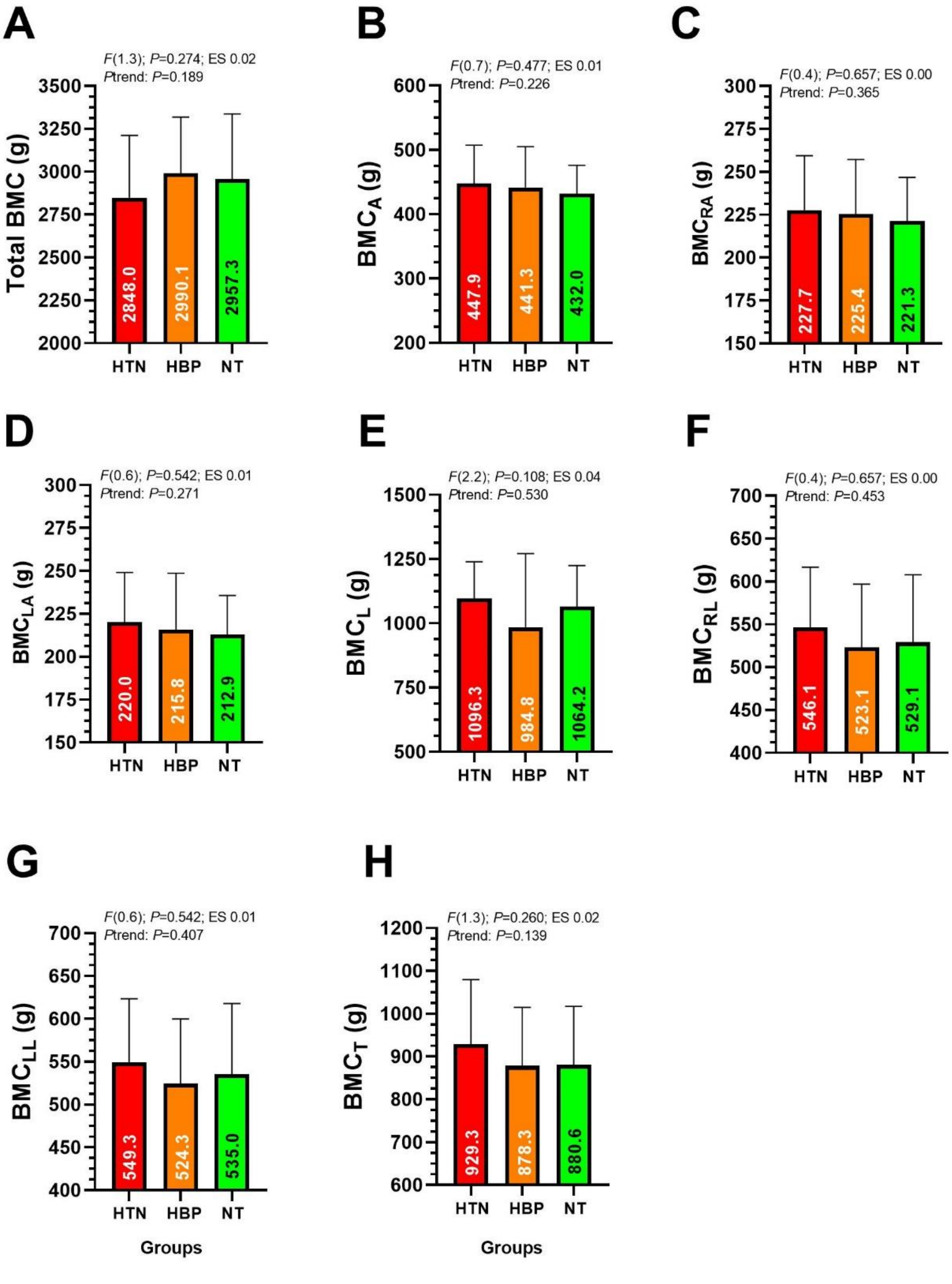

(Supplementary Material 2). Body composition characteristics by dual X-ray absorptiometry analysis (iDXA) with respect to bone mineral density (BMC) of type; total BMC (A), arm BMC (B), right arm BMC (C), left arm BMC (D), leg BMC (E), right leg BMC (F), left leg BMC (G) and trunk BMC (H) in Chilean diving workers with different levels of blood pressure. Groups are described as; (HTN) Hypertension, (HPB) High blood pressure and (NT) Normotensive. Data are analyzed by one-way ANOVA. (ES) Denotes Cohen's d effect size measure at $P<0.05$ level. (*P*trend) Denotes trend of the behavior of the values per group from HTN; HBP; and NT order).

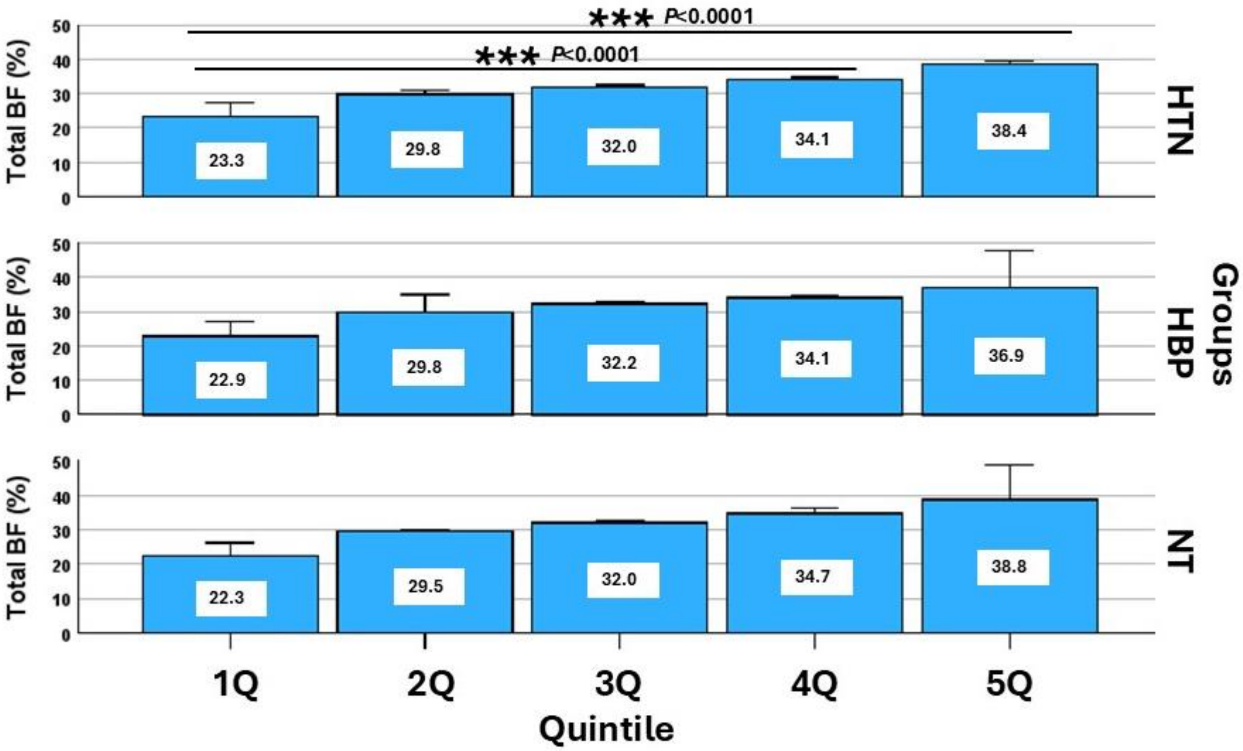

(**Supplementary Material 3**). Quintiles of body fat percentage by different blood pressure diving groups.

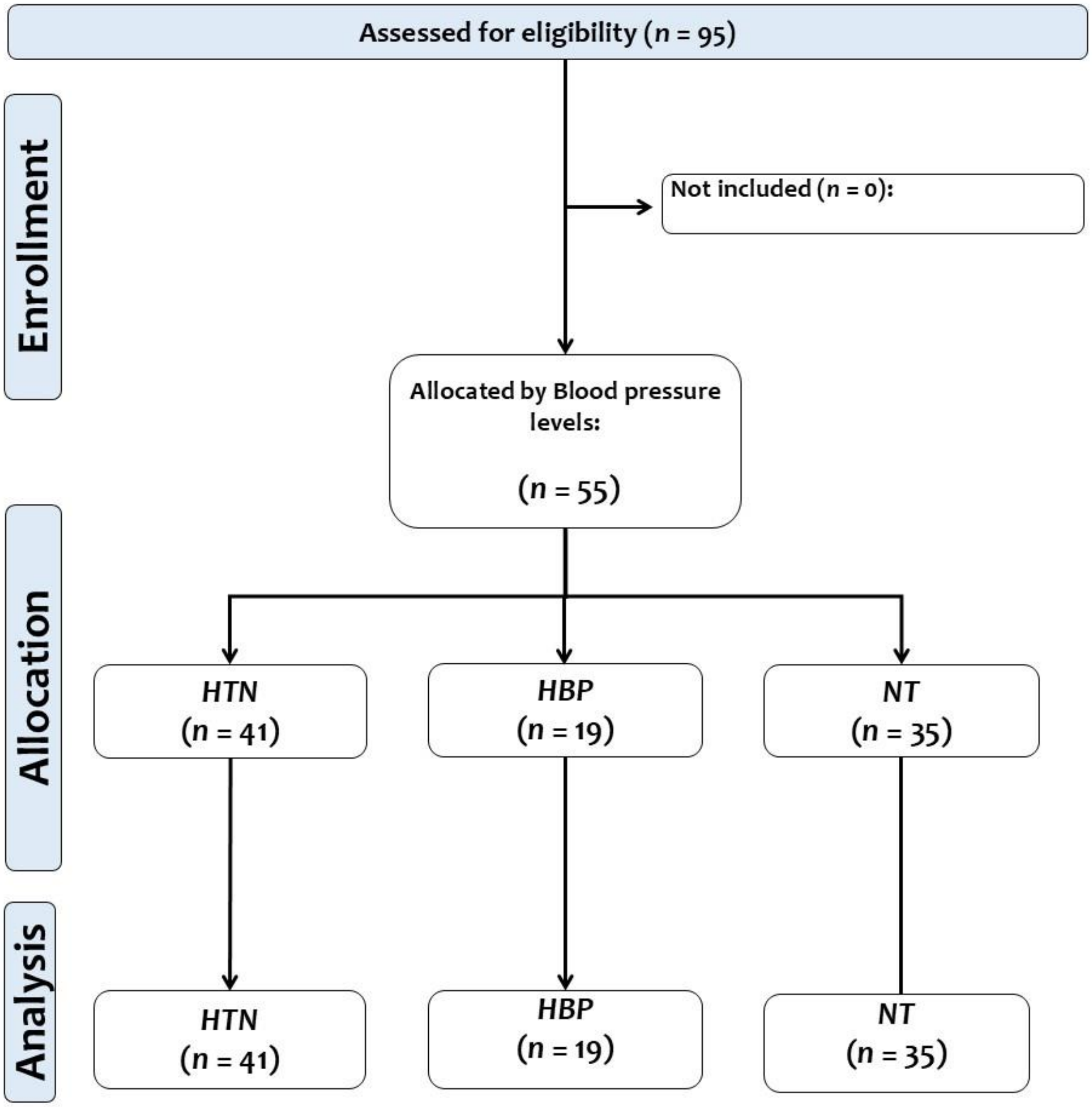

(**Supplementary Material 4)**. CONSORT study design.

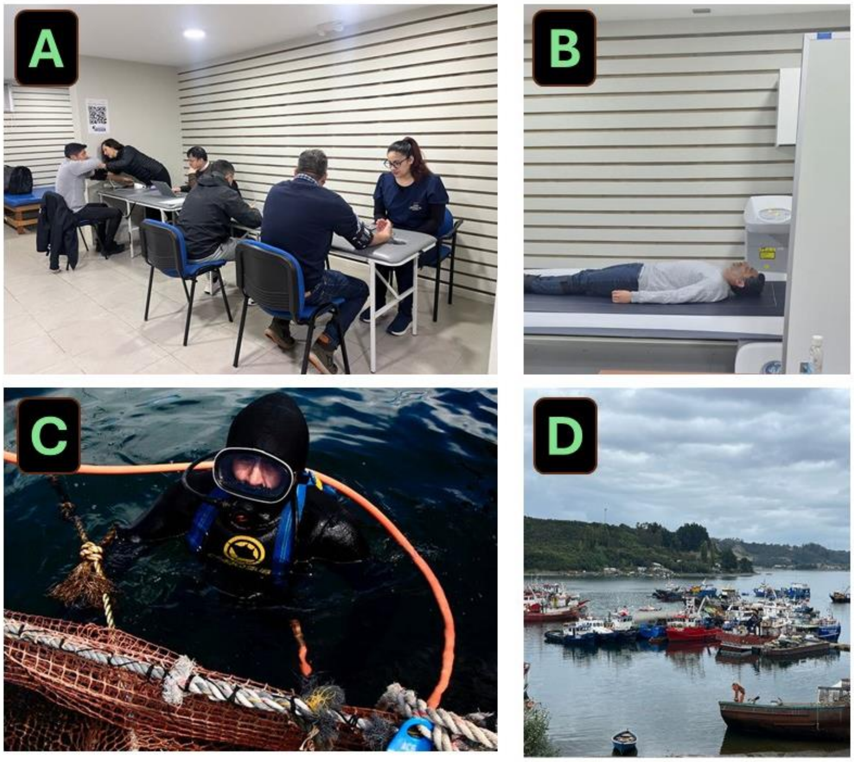

(**Supplementary Material 5).** Body composition iDXA measurements and characteristics of the diving equipment and conditions. Written informed consent was obtained from all participants and/or their legal guardians prior to study participation. All participants provided explicit authorization for the use and publication of any potentially identifiable information or images in this online open-access publication, in accordance with ethical standards and institutional guidelines.
